# Supplementary material for: Automated detection and staging of malaria parasites from cytological smears using convolutional neural networks
Source: Biol Imaging. 2021 Aug 2;1:e2. doi: 10.1017/S2633903X21000015 (PMC8724263; doi:10.1017/S2633903X21000015)
Supplement: Supplementary file 1 [file S2633903X21000015sup001.docx]

**Supplementary Information (SI)**

Automated detection and staging of malaria parasites from cytological smears using convolutional neural networks

#

# **Authors:** Mira S. Davidson^1^, Clare Andradi-Brown^1,2^, Sabrina Yahiya^1^, Jill Chmielewski^3^, Aidan J. O’Donnell^4^, Pratima Gurung^5^, Myriam Jeninga^6^, Parichat Prommana^7^, Dean Andrew^8^, Michaela Petter^6^, Chairat Uthaipibull^7^, Michelle Boyle^8^, George W. Ashdown^1^, Jeffrey D. Dvorin^5^, Sarah E. Reece^4^, Danny W. Wilson^3,9^, Kane A. Cunningham^10^, D. Michael Ando^11^, Michelle Dimon^11^ and Jake Baum^1*^

#

# *To whom correspondence should be addressed: Jake Baum, jake.baum@imperial.ac.uk

# **Short Title**: Machine learning for malaria parasite detection

**Supplementary Information**

Supplementary Figures S1-9

Supplementary Dataset S10

Supplementary Table S1-2


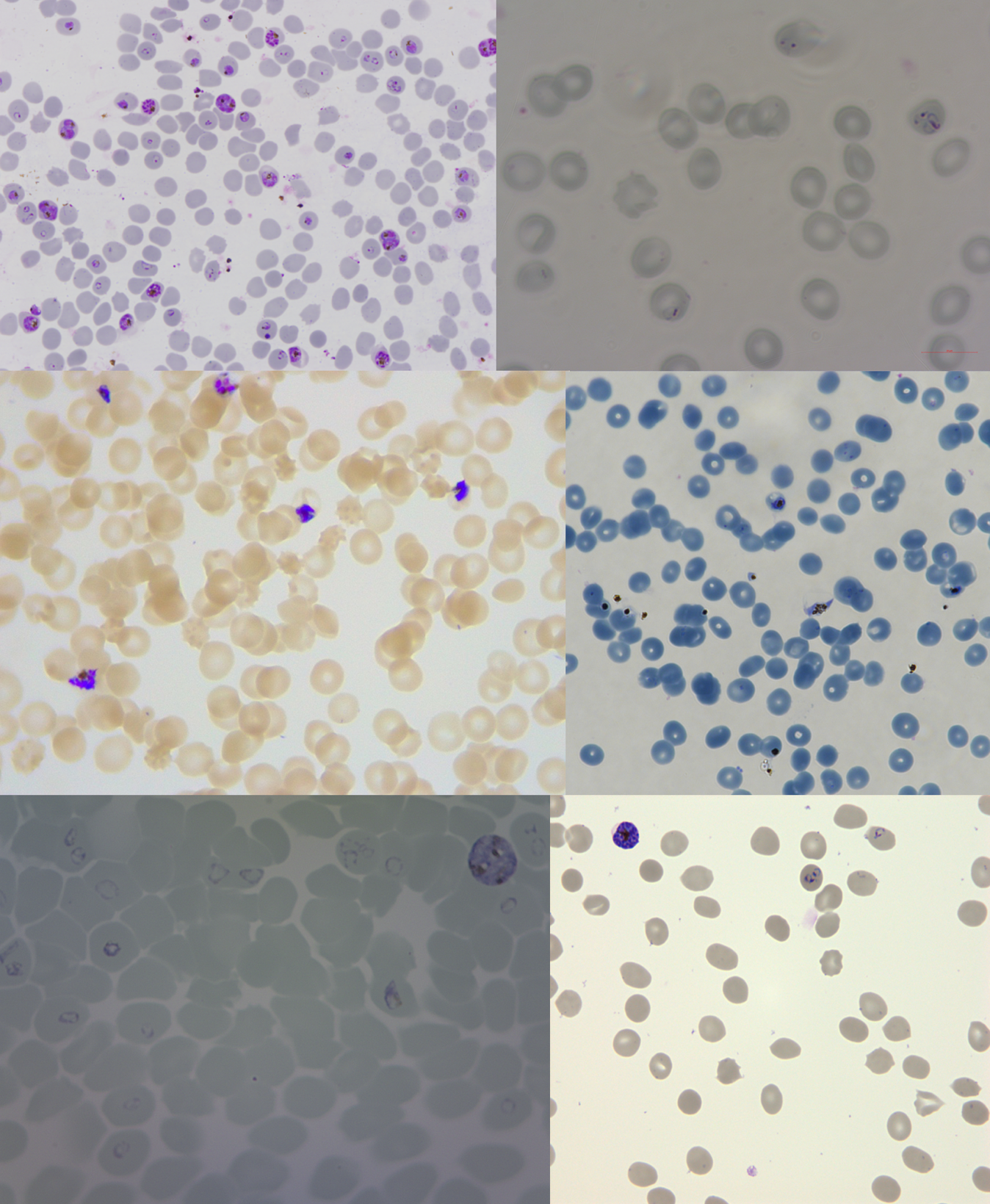


**Supplementary Figure S1**: **Example images from each dataset**. Images are in the same order as Supplementary Table S1 (left-to-right; top-to-bottom).


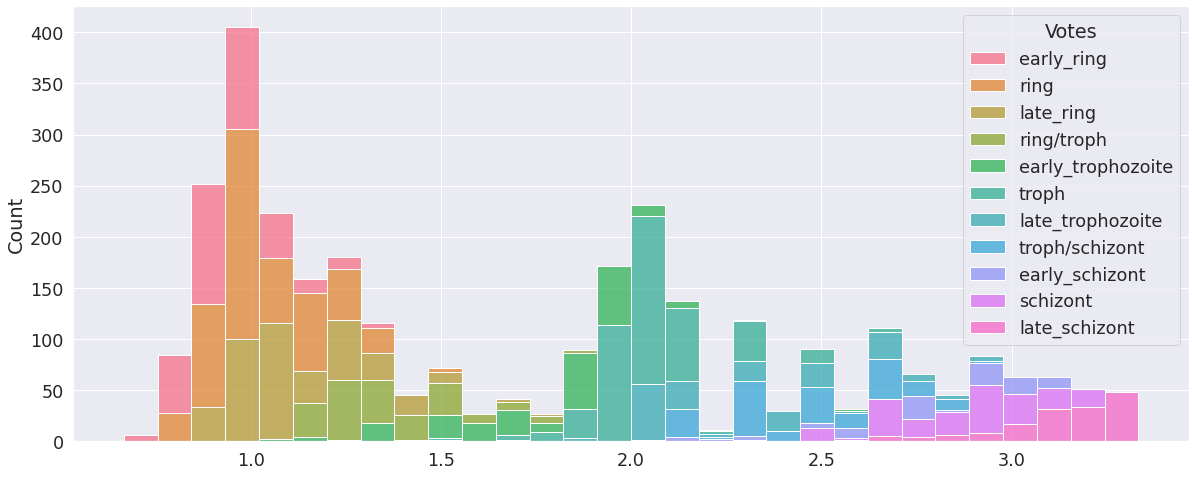


#### Supplementary Figure S2: Histogram of annotator labels for ground truth labels of intra-erythrocytic developmental cycle (IDC) stages. Annotators were shown an image of an RBC infected with a parasite from the IDC. Label options were early ring, late ring, early trophozoite, late trophozoite, early schizont, late schizont; a canonical ring would be annotated by selecting both “early ring” and “late ring”. Annotators were allowed to select multiple values. Labels were converted to a numeric with all labels assigned according to the scale of ring=1, trophozoite=2, schizont=3. All selected labels were merged and averaged to get a ground truth label, as presented on the x-axis. IDC stages are coloured according to individual labels from annotators.


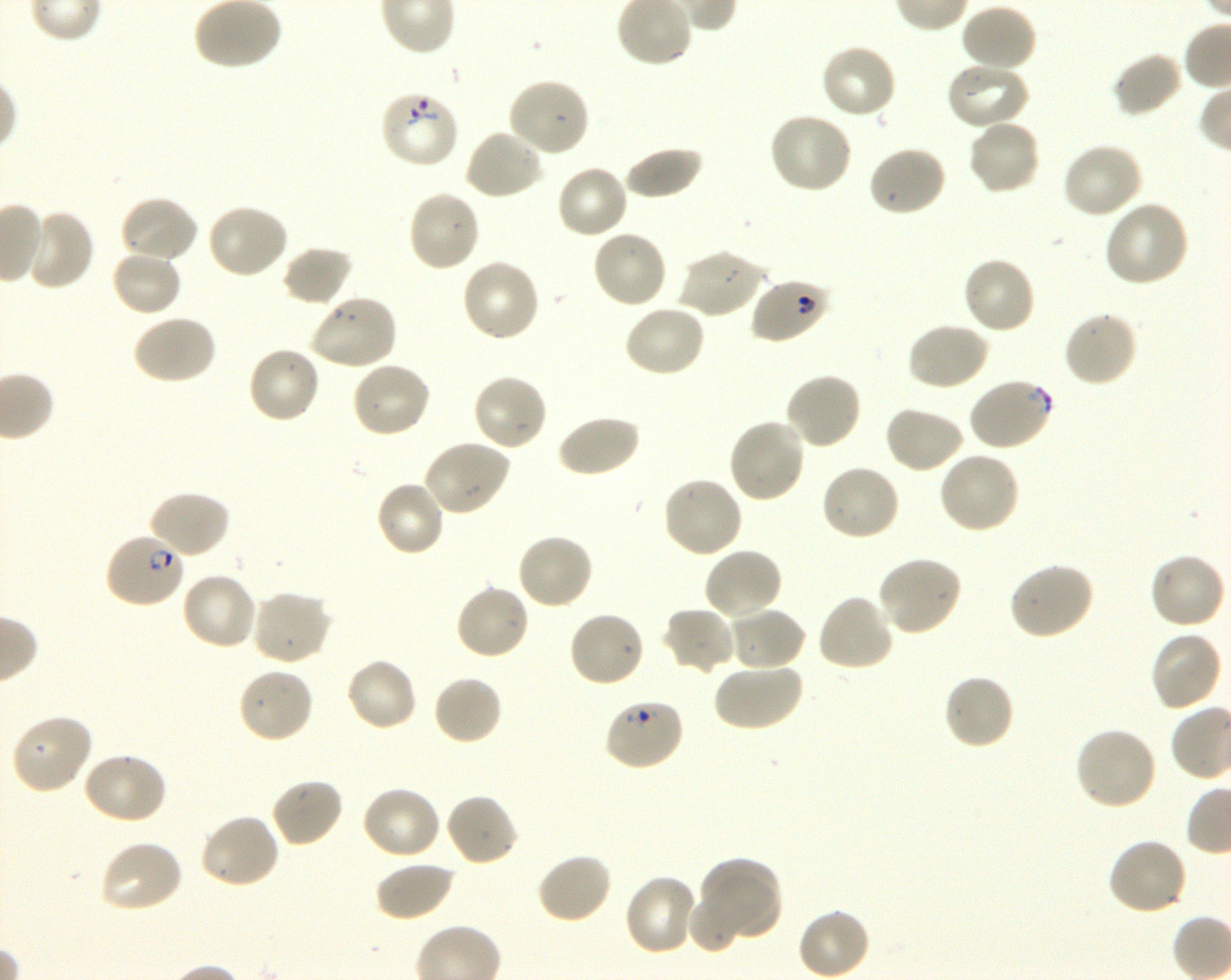


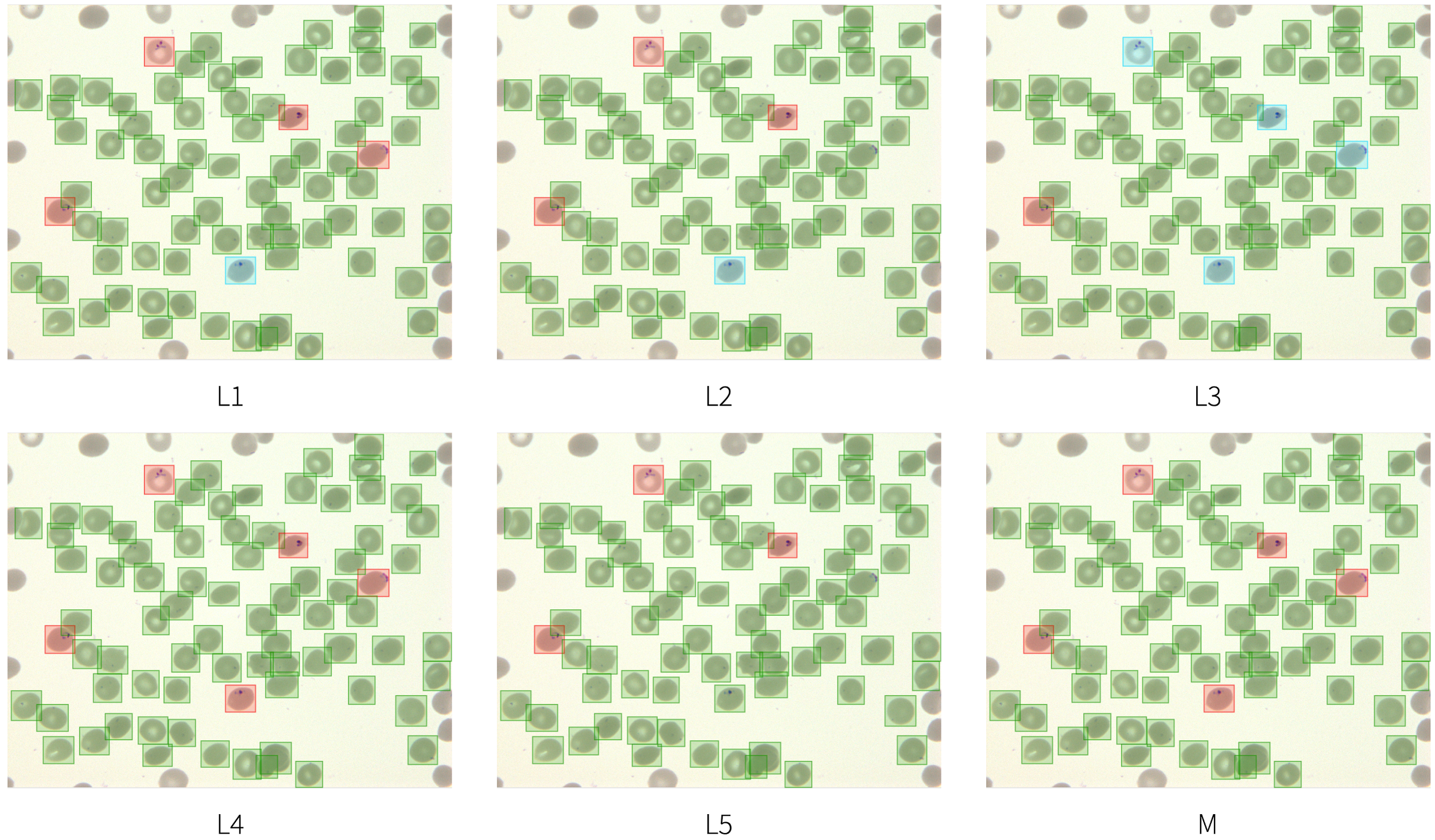


#### Supplementary Figure S3: Labelling variation in parasitaemia calculation. Five annotators (L1-5) across three different research centers were asked to correct bounding boxes around RBCs and label those infected. Only cells where all annotators agreed on infection were used for measuring performance. Uninfected cells (green), infected cells (red), and cells labelled as “unsure” (blue) are annotated. M=Model prediction.

**
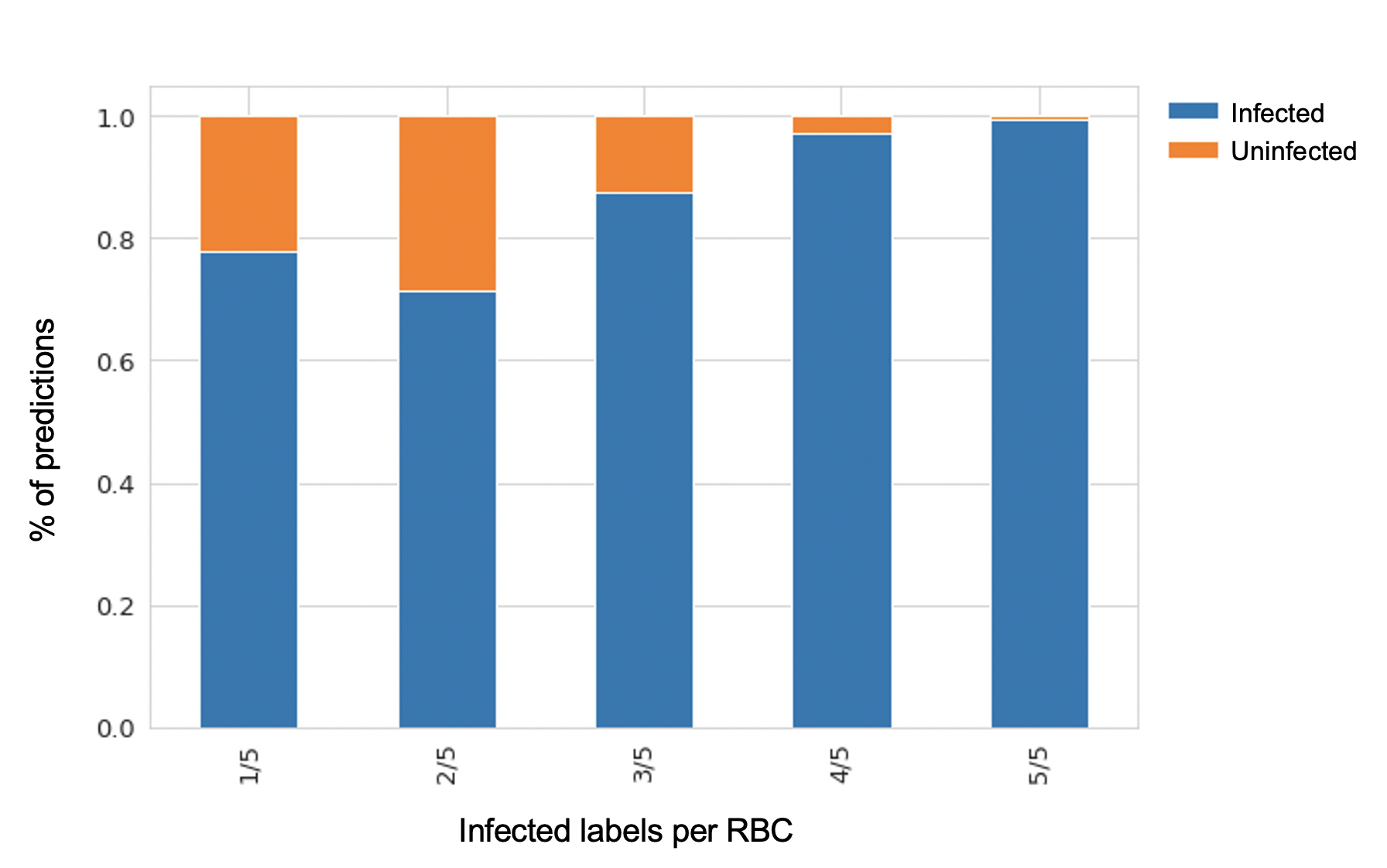
**

**Supplementary Figure S4: Percentage of predicted infected and uninfected RBCs against proportion of infected labels.**

**
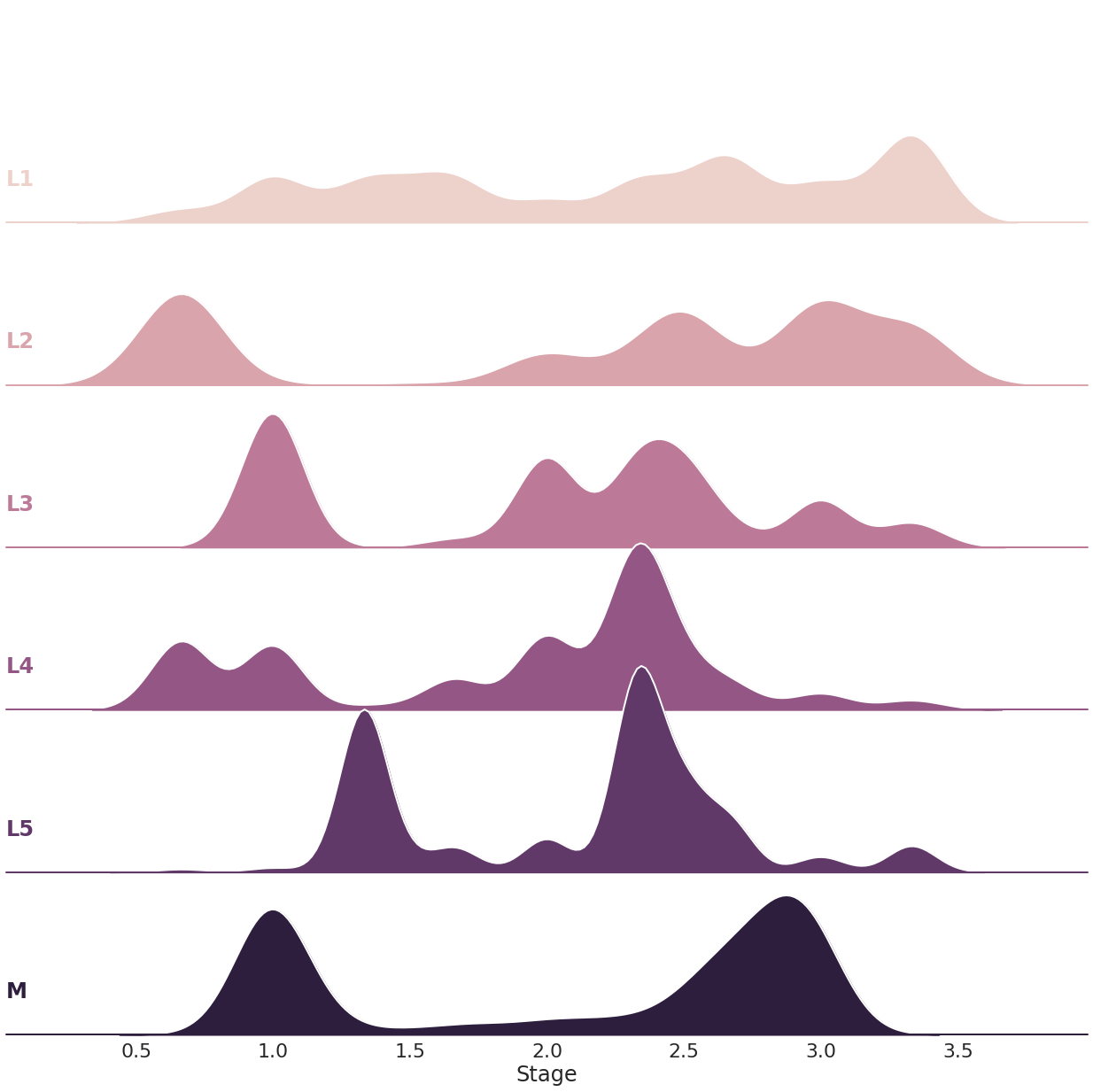
**

#### Supplementary Figure S5: Labelling variation in IDC life stage distribution. Five annotators (L1-5) across three different research centers were asked to label the same data set for segmented infected RBCs as a combination of early/late ring, trophozoite, and schizont labels. Labels were converted to a numeric scale where ring=1, trophozoite=2, schizont=3; e.g. a selection of late trophozoite and early schizont results in a value of 2.5. We observe large variability between annotators, highlighting the need for standardisation in the evaluation of blood smears and the potential of using automated methods towards this objective. M=Model prediction.

####
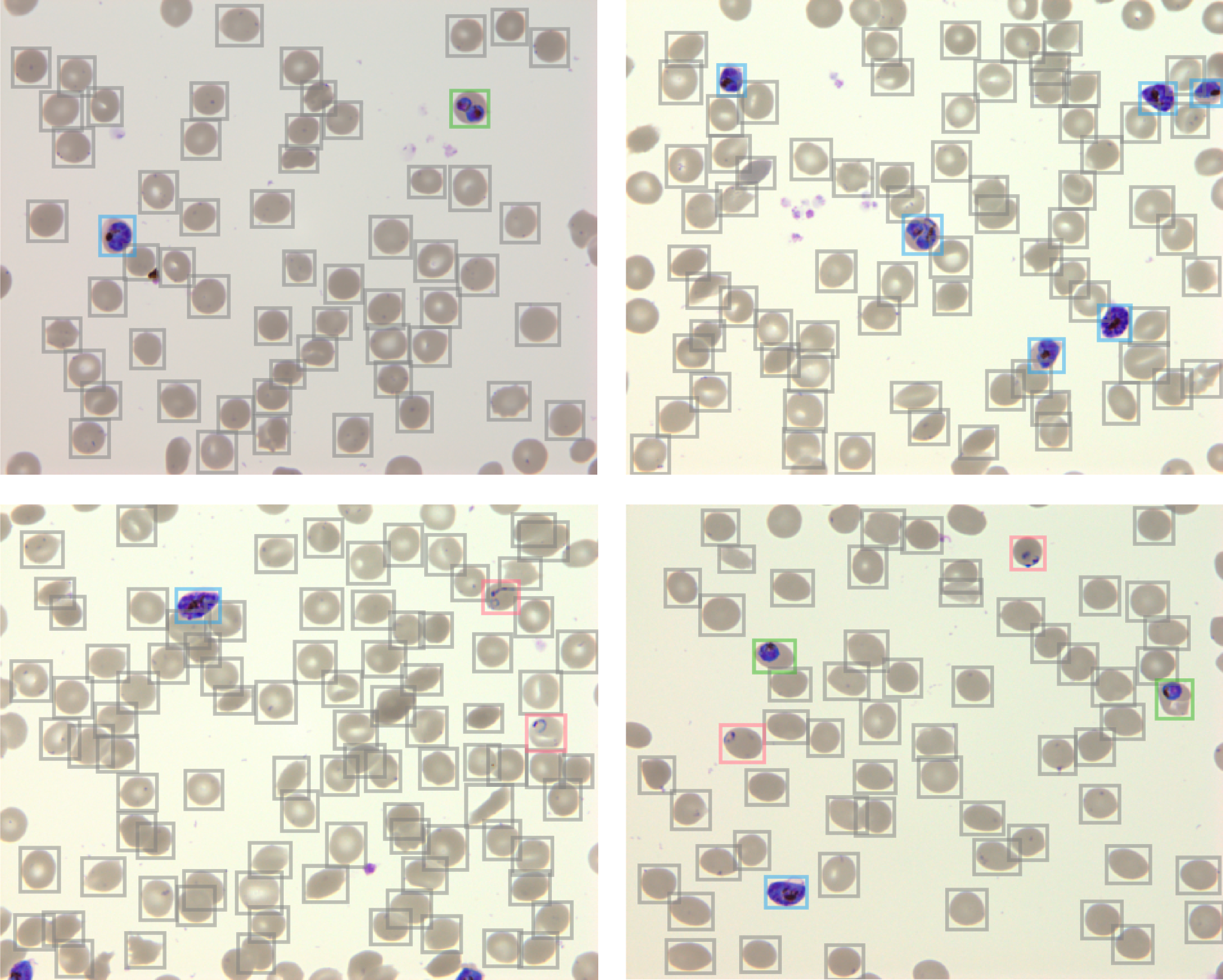


#### Supplementary Figure S6: PlasmoCount predictions on *P. falciparum* test set. Example images are provided as Supplementary Dataset S10 for testing purposes on [www.plasmocount.org](http://www.plasmocount.org) (login details please visit <https://www.baumlab.com/plasmocount>) and expected results are listed in Supplementary Table S2.

####
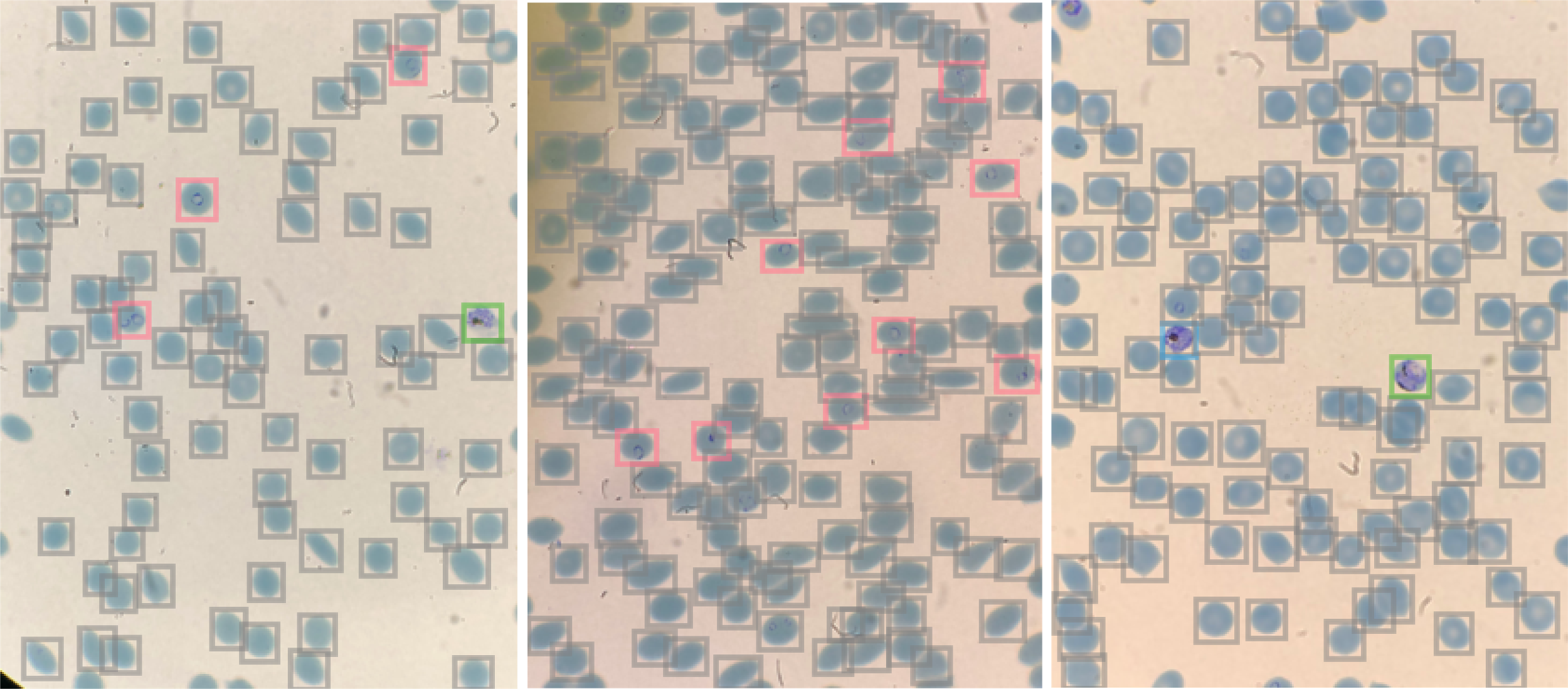


#### Supplementary Figure S7: PlasmoCount predictions using mobile phone pipeline. Images were captured with an iPhone 8+ camera at 2x zoom by aligning the camera with the microscope eyepiece (Nikon Ti2-E). Images were then uploaded to PlasmoCount for assessment of the blood films (ring=red, trophozoite=green, schizont=blue, uninfected RBC=grey).

####
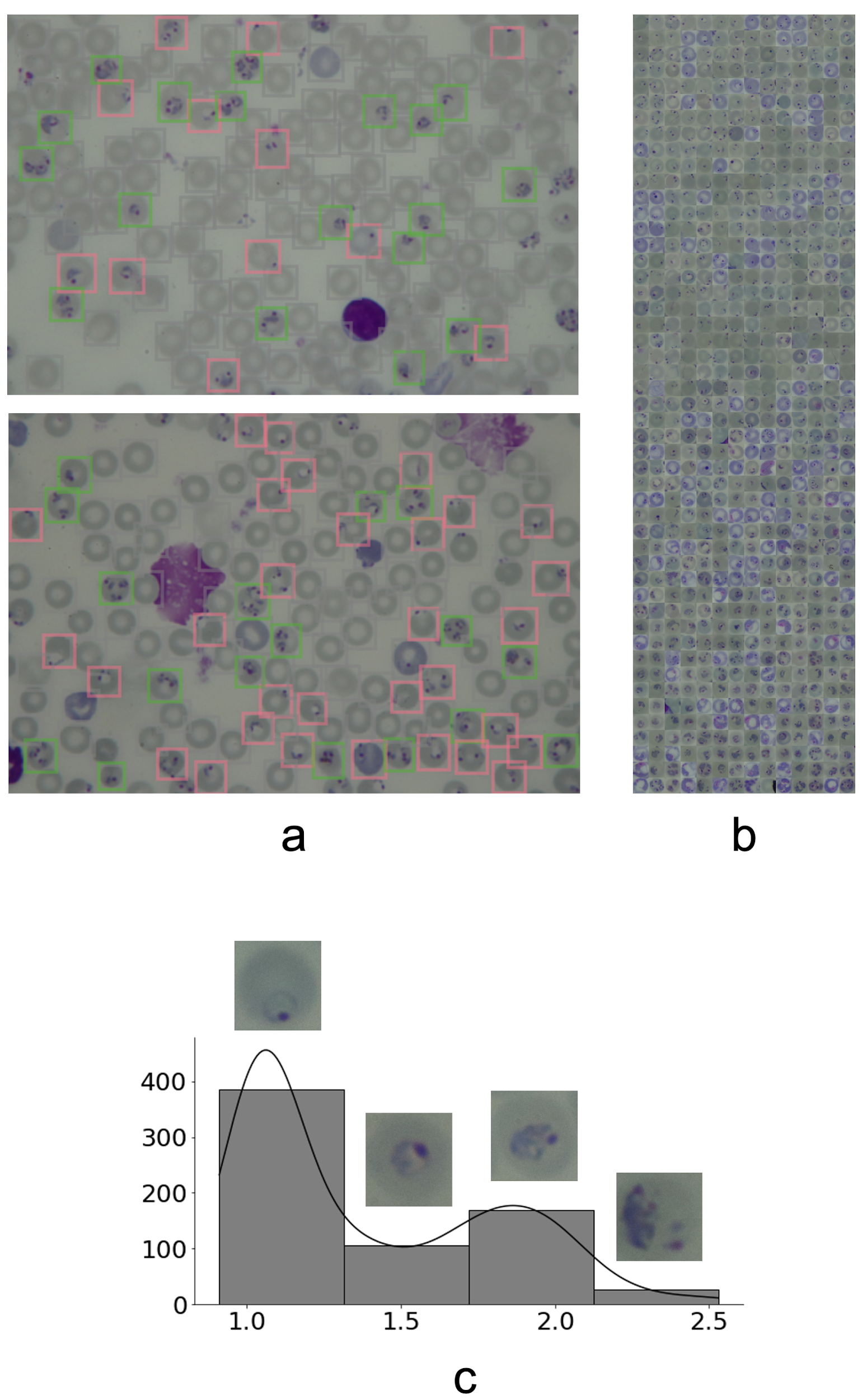


#### Supplementary Figure S8: PlasmoCount predictions on *P. chabaudi adami* clone DK data. Example images (a) show that using a per-image threshold for the area of bounding boxes can be sufficient to eliminate leukocytes and stain precipitation (ring=red, trophozoite=green, schizont=blue, uninfected RBC=grey). (b) Ordering of all detected infected RBCs by PlasmoCount (left to right; top to bottom). (c) Parasite distribution as predicted by PlasmoCount. Example RBCs are displayed for each bin. Bins shown generally correspond to ring, early trophozoite, mid trophozoite and late trophozoite stages according to our numeric scale (ring=1, trophozoite=2, schizont=3). A few gametocytes have been detected. These are not mistaken for rings and have been approximately placed in between mid trophozoites and schizonts. This suggests that future optimisation of the model may enable successful differentiation of IDC and gametocytes (which wasn’t attempted here) in other parasite species.

1. **Download and install python 3.9.5**

Download python at : <https://www.python.org/downloads/>

Ensure you can run python, in the command line type:

python3 --version

1. **Download and install Yarn v3.00 and Node.js**

It is recommended to install Yarn and Node.js together through the npm package manager.

1. **Install npm and Node.js**

npm is distributed with Node.js

Download both at : <https://nodejs.org/en/>

To confirm you have Node.js, in the command line run:

node -v

To confirm you have npm installed, in the command line run:

npm -v

1. **Install Yarn**

Once you have installed npm run the following in the command line to install Yarn:

npm install –global yarn

To confirm you have Yarn installed, in the command line run:

yarn --version

**3. Install pip and python dependencies**

The recommended way to install Python library dependencies is with the pip command.

1. **Check pip is installed**, in the command line run

pip --version

If pip is not installed visit: <https://packaging.python.org/tutorials/installing-packages/#ensure-you-can-run-pip-from-the-command-line>

and go to section: Ensure you can run pip from the command line

**b. Install Python dependencies using pip**

requirements.txt is available at PlasmoCount/api/requirements.txt

and contains all the Python dependencies required

On the command line run:

pip install -r api/requirements.txt

**4. Install Node.js dependencies**

On the command line run

npm install

This will install all modules listed as dependencies and devDependencies in the package.json file (Plasmocount/package.json).

**5. Install Yarn dependencies**

On the command line run

yarn install

to install all Yarn dependencies in the package.json file (Plasmocount/package.json).

**Supplementary Figure S9: Guide to download and install dependencies for PlasmoCount.**

#### Supplementary Table S1: Dataset specifications. Test set is highlighted.

| **Microscope brand +**  **model** | **Lens objective (x)** | **Numerical aperture** | **Malaria species + strain** | **Parasitemia** | **Donor Blood Group** | **Cultivation** | **# images / # objects** |
| --- | --- | --- | --- | --- | --- | --- | --- |
| Olympus | 100 | 1.4 | *Plasmodium falciparum* 3D7 | 20% | O | Static culture | 20 / 4414 |
| Zeiss Axioskop 40 | 100 | 1.25 | *Plasmodium falciparum* NF54 | 10% | A+ | Static culture | 66 / 2397 |
| Leica DM750 | 100 | 1.25 | *Plasmodium falciparum* 3D7 | 4.4% | O+ | Static culture | 65 / 9869 |
| Nikon Ti2-E Inverted Microscope | 100 | 1.45 | *Plasmodium falciparum* NF54 | >2% | A+/O+ | Static culture | 84 / 8552 |
| Olympus LC20 | 100 | 1.25 | *Plasmodium falciparum* 3D7, DD2, D10 | 4-6% | O+ | Static culture | 48 / 3228 |
| Olympus BX40 with INFINITY3-6UR camera | 100 | 1.30 | *Plasmodium falciparum 3D7* | 3% | O+ | Shaking culture | 121 / 9067 |

**Supplementary Table S2: PlasmoCount results for Supplementary Dataset S10.**

| **Name** | **# cells** | **Parasitaemia** | **#R** | **#T** | **#S** | **#G** |
| --- | --- | --- | --- | --- | --- | --- |
| 1.jpg | 58 | 0.03 | 0 | 1 | 1 | 0 |
| 2.jpg | 77 | 0.08 | 0 | 0 | 6 | 0 |
| 3.jpg | 92 | 0.03 | 2 | 0 | 1 | 0 |
| 4.jpg | 61 | 0.08 | 2 | 2 | 1 | 0 |
| 5.jpg | 82 | 0.05 | 3 | 1 | 0 | 0 |
| 6.jpg | 135 | 0.07 | 9 | 0 | 0 | 0 |
| 7.jpg | 100 | 0.02 | 0 | 1 | 1 | 0 |
| 8.jpg | 137 | 0.22 | 12 | 18 | 0 | 0 |
| 9.jpg | 131 | 0.37 | 31 | 17 | 0 | 0 |
